# Innate Resistance to *Leishmania amazonensis* Infection in Rat Is Dependent on NOS2

**DOI:** 10.3389/fmicb.2021.733286

**Published:** 2021-10-29

**Authors:** Yun-Fu Chen, Si-Fei Yu, Chang-You Wu, Na Wu, Jia Shen, Juan Shen, Jiang-Mei Gao, Yan-Zi Wen, Geoff Hide, De-Hua Lai, Zhao-Rong Lun

**Affiliations:** ^1^Guangdong Provincial Key Laboratory of Aquatic Economic Animals, Key Laboratory of Gene Engineering of the Ministry of Education, State Key Laboratory of Biocontrol, School of Life Sciences, Sun Yat-Sen University, Guangzhou, China; ^2^Institute of Immunology and Key Laboratory of Tropical Disease Control of the Ministry of Education, Zhongshan School of Medicine, Sun Yat-Sen University, Guangzhou, China; ^3^Ecosystems and Environment Research Centre and Biomedical Research Centre, School of Science, Engineering and Environment, University of Salford, Salford, United Kingdom

**Keywords:** nitric oxide, Leishmania (L.) amazonensis, rat peritoneal macrophages, iNOS knock out, adoptive transfer

## Abstract

*Leishmania* infection causes diverse clinical manifestations in humans. The disease outcome is complicated by the combination of many host and parasite factors. Inbred mouse strains vary in resistance to *Leishmania major* but are highly susceptible to *Leishmania amazonensis* infection. However, rats are highly resistant to *L. amazonensis* infection due to unknown mechanisms. We use the inducible nitric oxide synthase (*Nos2)* gene knockout rat model (*Nos2*^−/−^ rat) to investigate the role of NOS2 against leishmania infection in rats. Our results demonstrated that diversion toward the NOS2 pathway is the key factor explaining the resistance of rats against *L. amazonensis* infection. Rats deficient in NOS2 are susceptible to *L. amazonensis* infection even though their immune response to infection is still strong. Moreover, adoptive transfer of NOS2 competent macrophages into *Nos2*^−/−^ rats significantly reduced disease development and parasite load. Thus, we conclude that the distinct L-arginine metabolism, observed in rat macrophages, is the basis of the strong innate resistance to *Leishmania*. These data highlight that macrophages from different hosts possess distinctive properties and produce different outcomes in innate immunity to *Leishmania* infections.

## Introduction

Leishmaniasis, caused by *Leishmania* spp., is responsible for a wide spectrum of human diseases ranging from cutaneous leishmaniasis (CL), through mucocutaneous leishmaniasis (MCL) to the fatal form of visceral leishmaniasis (VL; Murray et al., 2005). It is endemic in 98 countries rendering approximately 350 million people at risk of infection worldwide (World Health Organization, 2010). *Leishmania* species are obligate intracellular protozoan parasites in the mammalian host and reside as aflagellated amastigote forms that primarily infect phagocytes, but exist as flagellated promastigotes in the gut of infected sandflies (Kaye and Scott, 2011).

The outcome of leishmaniasis is governed by both host and parasite factors (Alexander et al., 1999; Kaye and Scott, 2011). Experimental models of leishmaniasis have provided a large amount of useful data which have helped to elucidate the host immune defense mechanism against infection. Mouse models have been extensively exploited to investigate the host factors that might be responsible for resistance or susceptibility to infection. For instance, most inbred mouse strains are highly susceptible to *L. amazonensis* infection (Barral et al., 1983; Calabrese and da Costa, 1992; Soong et al., 1997). However, the best recognized model is the *Leishmania major* model of cutaneous leishmaniasis where inbred strains of mice vary in their ability to control *L. major* infection. For example, in BALB/c mice, susceptibility is due to the development of a dominant type 2 helper T-cell (Th2) suppressive immune response, while in resistant C57BL/6 mice, they are able to mount an effective Th1 response. This means that host resistance/susceptibility is influenced by expansion of distinct helper T-cell subsets that are followed by the production of divergent cytokine profiles and macrophage function (Muller et al., 1989; Sacks and Noben-Trauth, 2002). Macrophages comprise the majority of *Leishmania-*infected cells and are the main effector cells that decide the fate of intracellular amastigotes. In the context of a Th1 response, macrophage inducible nitric oxide synthase (NOS2) is activated to produce nitric oxide (NO), resulting in the destruction of intracellular amastigotes. However, in the Th2 response, alternatively activated macrophages upregulate arginase expression that suppresses NOS2 activity and leads to uncontrolled parasite proliferation (Liew et al., 1991; Stenger et al., 1994; Wei et al., 1995; MacMicking et al., 1997). The hamster model is another frequently used model for leishmaniasis because it has clinical manifestations that closely mimic that of humans. Hamsters are highly susceptible to *Leishmania* infection due to the lack of a binding sequence for nuclear factor interleukin-6 (NF-IL-6) within the *Nos2* promoter, rendering it refractory to activation by IFN-γ/LPS, and resulting in impaired NOS2-mediated intracellular killing (Melby et al., 2001; Perez et al., 2006; Saldarriaga et al., 2012).

In a remarkable contrast to mice and hamsters, rats (*Rattus rattus* and others) in the laboratory are highly resistant to *Leishmania* infection (Giannini, 1985). Rats are frequently used as animal models in many biological studies (e.g., physiology, transplantation, medicine, and cancer) as they differ in many aspects when compared with mice. Very limited experimental work has been reported on the use of rats for investigating *Leishmania* infection. In the choice of an animal model to investigate host immune defense mechanisms against pathogens, the rat model is usually overlooked in favor of the mouse model. This probably results from the following facts: 1. Mice are generally susceptible to many infectious agents and easy to establish infection. 2. Transgenics and knockouts in mice are generally easy to produce, and there are multiple genetically engineered mice that are already available worldwide. 3. Immunological reagents and tools are readily available for mice while somewhat limited in rats. However, rats are generally more resistant than mice to many pathogen infections – thus offering an opportunity to explore mechanisms of resistance. This host difference is commonly, and traditionally, explained by host specificity but generally these explanations lack a clearly identified factor or process. It suggests that there should be striking differences in the immune response (innate or adaptive) between rats and mice during pathogen infection. Therefore, elucidation of the mechanisms of rat resistance to pathogen infection would provide an important impact on our understanding of the differences in the innate immunity between rats and mice.

In this study, a rat model of *Leishmania amazonensis* infection has been used to investigate the rat determinants that drive resistance to *Leishmania* infection. *Leishmania* is an obligate parasite that infects macrophage lineages, thus providing an excellent model to focus on macrophage function during infection. *L. amazonensis* was chosen because it is one of the most virulent species in the genus *Leishmania* and possesses powerful mechanisms to subvert the host protective immune response (Soong, 2012). In this work, we demonstrate that rat macrophages possess a significantly different L-arginine metabolism from mouse macrophages. This is characterized by the predominant usage of L-arginine by the NOS2 pathway, which confers strong resistance to *Leishmania* infection.

## Materials and Methods

### Animals

Female Sprague Dawley (SD) rats, BALB/c mice, and C57BL/6 mice were purchased from Zhongshan School of Medicine, Sun Yat-Sen University (SYSU), Guangzhou. Female *Nos2*^−/−^ SD rats were generated by TALENs and bred in the animal facility of SYSU (Shen et al., 2017). All the animals were maintained in specific pathogen-free conditions. Protocols for the use of animals were approved by the Institutional Review Board for Animal Care at Sun Yat-Sen University (NO:31472058).

### Antibodies and Reagents

Monoclonal antibodies used for phenotypic and intracellular cytokine analyses: isothiocyanate (FITC)-anti-CD3, PE-Cy7-labeled anti-CD4, and peridinin-chlorophyll protein (PerCP)-labeled anti-CD8 were purchased from BD Biosciences (San Jose, CA). Rat ELISA kits for cytokine IFN-γ and IL-4 were purchased from BD Biosciences.

### Parasites and Culture

*Leishmania amazonensis* LV78 (RAT/BA/74/LV78) causes only cutaneous infection, a clone expressing enhanced green fluorescent protein (eGFP) was kindly provided by Dr. K. P. Chang. This parasite showed no difference in infection virulence to macrophages or mice compared to the wild-type strain (Chan et al., 2003). The amastigotes of the LV78 strain were freshly isolated from footpad lesions of previously infected BALB/c mice and transformed into promastigotes in Schneider’s insect medium (SDM, pH 6.9) supplemented with 10% FBS (Hyclone) at 26°C. The parasites at stationary phase (day 5 culture) were used for *in vitro* or *in vivo* infection. Only 2 to 9 passages were used in subculture of the parasites in order to make sure that virulence of the parasites was maintained for infection.

### Isolation of Peritoneal Macrophages and Infection With *Leishmania*

Naïve peritoneal macrophages were isolated by peritoneal lavage with ice-cold D-hanks buffer as described preciously (Li et al., 2012). Briefly, WT and *Nos2*^−/−^ rats (females, 9weeks old) were sacrificed by CO_2_ asphyxia. A total of 20ml ice-cold D-hanks buffer were injected into the peritoneal cavity and then withdrawn. Cells were collected by centrifugation and resuspended in RPMI-1640 (without phenol red) supplemented with 10% FBS and 100U/ml penicillin and streptomycin. Then, 5×10^5^ macrophages were seeded into each well of a 24-well plate with 500μl medium. Four hours later, unattached cells were removed by washing twice with pre-warmed D-hanks buffer to obtain macrophages. Macrophages were allowed to culture overnight before infection. LV78 promastigotes from stationary culture were washed 3 times in D-hanks buffer, resuspended in complete RPMI-1640 medium, and adjusted to a final concentration of 2.5×10^7^. Aliquots of 100μl were put into each well of a 24-well plate with a ratio of parasites to macrophages of 5:1. They were then incubated at 35°C with 5% CO_2_ for 6h, and free promastigotes were removed by washing 3 times with pre-warmed D-hanks. Cells were treated with or without various combinations of IFN-γ (50ng/ml) and aminoguanidine (AG, 5mM). Culture supernatants were collected at indicated time points to determine the production of NO. At 0, 24, 48, and 72h postinfection, and parasite proliferation was determined by counting at least 300 macrophages to enumerate the percentage of cells infected and amastigotes per 100 macrophages.

### Determination of Nitric Oxide Production

Culture supernatants were collected from treated cells. Proteins were removed by adding ZnSO_4_ to a final concentration of 30mg/ml, and the liquid was thoroughly vortexed and centrifuged at 8,000g for 10min. Then, 100μl of total supernatant was mixed with 100μl Griess reagent and incubated in the dark for 10min; the absorbance was read at 540nm, and nitrite concentrations were calculated against a standard curve.

### Determination of Arginase Activity

Arginase activity of macrophages was measured by a colorimetric method as described (Corraliza et al., 1995). Tenmm MnCl_2_ and 0.5ML-arginine were successively added to macrophage lysates for 1h at 37°C. The reaction was stopped by addition of an acid solution (H_2_SO_4_: H_3_PO_4_: H_2_O=1:3:7), and the urea generated by arginase was analyzed by addition of a-isonitrosopropiophenone at 100°C for 45min. The colored product was quantified by absorption at 550nm in an ELISA reader. Arginase activity was determined as the amount of urea produced from total protein of peritoneal macrophages.

### Infection of Animals and Determination of Lesion Development

Groups of WT, heterozygous (Het), and *Nos2*^−/−^ SD rat (*n*=12, in each group, 4weeks old) were subcutaneously infected in the left ear with 10^7^ stationary phase *L. amazonensis* LV78 promastigotes, using a 30G needle, in a volume of 10μl PBS, while the control rat ear received 10μl PBS without parasites. Uninfected rats of the same age were used as controls. The lesion thickness and diameter were measured every 4 or 8days with a dial caliper. Lesion thickness was calculated by subtracting the thickness of the control ear from the infected ear, while lesion diameter was measured as the longest axis of the ear lesion.

### Estimation of Parasite Load by Limiting Dilution Assay

At 32 and 64days postinfection, 3 rats per group were sacrificed and the parasite load in the infected ears was estimated by using a limiting dilution assay as previously described with modification (Belkaid et al., 2000). Briefly, the infected ears were cutoff, and their surfaces were disinfected in 70% ethanol, and the dorsal and ventral part of the ear were separated with forceps. The two ear leaflets were placed dermal side down on SDM containing 1mg/ml collagenase 1 (Sigma) and digested for 2h in 37°C. Then, the dermal sheets were cut into small pieces and homogenized in a glass tissue homogenizer with 2ml SDM followed by the liquid being passed through a 70μm cell strainer (Falcon, BD) to obtain a single cell suspension. Serial 10-fold dilutions were made with SDM supplemented with 10% FBS and 5% defibrinated rabbit whole blood lysate, and aliquots of 100μl were distributed in 96-well plates with 12 wells per dilution. The plates were kept at 26°C for up to two weeks, and the number of parasites was determined by the highest dilution for which the parasites could be grown.

### Histopathology

Infected ears were collected and fixed in 4% paraformaldehyde for 24h. They were dehydrated with gradient solutions of ethanol and embedded in paraffin. The tissue specimens were sliced to a thickness of 5μm and stained with hematoxylin and eosin. All histological sections were observed by light microscopy (Leica DM 2500B, Germany).

### Preparation of Soluble *Leishmania* Antigen

*L. amazonensis* LV78 promastigotes were grown to stationary phase in SDM and were collected and washed three times in PBS and then resuspended in PBS at 10^9^/ml. The cells were subjected to 10cycles of rapid freezing (−70°C) and thawing at (37°C) for lysis and centrifuged at 12,000rpm at 4°C for 10min, and the supernatants were then collected and passed through a 0.20-μl filter assembly. The protein concentration was determined by a BCA kit. SLA was stored at −70°C until use.

### Analysis of Immune Cell Responses

At 32days postinfection, immune cells from *Leishmania*-infected WT and *Nos2*^−/−^ SD rats were isolated for analysis. PBMCs were isolated by Ficoll-Hypaque (Tianjin Hao Yang Biological Manufacture, Tianjin, China) density gradient centrifugation. Briefly, cells were suspended in Hank’s buffer and gently added on top of the Ficoll solution, and the ratio of cell suspension to Ficoll was 4:3 and centrifuged at a speed of 2,200rpm for 20min. The cells in the second upper layer were harvested for mononuclear leukocytes and washed twice with D-Hanks. Spleen and lymph nodes cells were separated by traditional methods. Briefly, the spleens and mesenteric lymph nodes from rats were mashed to release the cells and they were then passed through a 70μm cell strainer to obtain single cells. Red blood cells were removed using ammonium chloride as a lysing reagent, and immune cells were then washed with D-Hanks buffer and resuspended in RPMI-1640 supplemented with 10% FBS. Cells were counted and seeded into each well of a 96-well plate with 2× 10^5^ cell/ml. Cells were stimulated with SLA (25μg/ml) for 48h. IFN-γ and IL-4 production in the supernatants were determined by ELISA.

### Cytokine ELISA

Supernatant from SLA-stimulated cells were assayed for IFN-γ and IL-4 with rat ELISA kits (BD Biosciences). Absorbances were read at 405nm using a microtiter plate reader. Concentrations were determined using standard curves for each cytokine. The detection limits of IFN-γ and IL-4 assay kits were 31.25 and 1.6pg./ml, respectively.

### Serum Cytokine Analysis

Rat serum was collected at 16, 48, and 64days postinfection, and cytokines were analyzed by Multiplex Luminex using ProcartaPlex Rat Th Complete Panel (ProcartaPlex, eBioscience), according to the manufacturer’s instructions.

### Isolation of Rat Bone Marrow-Derived Macrophages and Transfer to Infected *Nos2*^−/−^ Rats

Bone marrow-derived macrophages (BMDMs) were generated as previously described with modification (Boltz-Nitulescu et al., 1987). Briefly, bone marrow cells were obtained by flushing femurs of adult SD rats with D-Hanks buffer, cells were then dispersed by passing through a needle, and erythrocytes were removed by lysis with 0.015M Tris-NH_4_Cl (pH 7.4). The cells were then washed and resuspended in DMEM supplemented with 10% FBS and 30% L929 cell supernatant. The cells were cultured in a 75cm^2^ TC flask for 3days with a change in fresh medium for another 4days. Then, the BMDMs were harvested by digestion with 0.5% trypsin containing 0.03% EDTA. The harvested BMDMs were washed 3 times in PBS and adjusted to 10^7^ cells/ml in PBS. The cells were i.v. injected into the recipient *Nos2*^−/−^ rat at 0, 8, 16, and 24 DPI.

### Statistical Analyses

Statistical differences between groups were analyzed with Student’s t test using a built-in module of GraphPad Prism v5.0 (GraphPad software), using *p*<0.05 as the level of significance.

## Results

### NOS2 Activity Is Essential for Rat Peritoneal Macrophage Resistance Against *L. amazonensis* Infection

**Figure 1:**
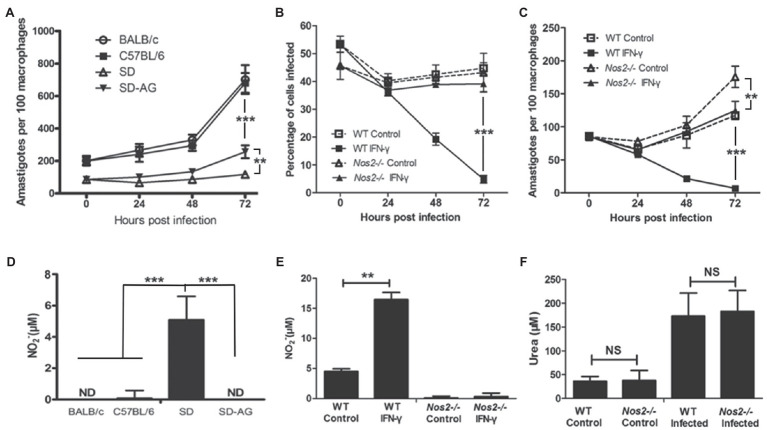
NOS2 activity is indispensable for rat macrophage resistance to *L. amazonensis* infection *in vitro*. **A,C**, Peritoneal macrophages from BALB/C, C57BL/6 mice, wild-type SD rats (SD, WT), or *Nos2*^−/−^ SD rats (*Nos2*^−/−^) were incubated with *L. amazonensis* promastigotes, with 5mm aminoguanidine (AG) or 50ng/ml recombinant rat IFN-γ, if indicated. The number of amastigotes per 100 macrophages **(A,C)** and percentage of macrophages infected **(B)** were shown. **(D,F)**, Corresponding NO production **(D,E)** and urea production **(F)** in culture supernatants at 24h postinfection were determined. The data (mean±SD amastigotes per macrophage) were derived from a single representative of three independent experiments. ^**^indicates a significant difference (0.001 ﹤ *P* ﹤ 0.01), ^***^(*P* ﹤ 0.001).

To investigate the mechanism of rat resistance against *Leishmania* infection, macrophages were targeted since they play a crucial role in the host innate immune response and are the major cell type inhabited by *Leishmania* during infection (Rittig and Bogdan, 2000). Naïve peritoneal macrophages were isolated from mice and Sprague Dawley (SD) rats and infected with *L. amazonensis in vitro*. In accordance with previous studies (Giannini, 1985; Soong et al., 1997), macrophages from both BALB/c and C57BL/6 mice are susceptible to *L. amazonensis* infection as indicated by unrestrained intracellular parasite replication during infection and by NO being absent in the culture supernatant (Figures 1A,D). In contrast, macrophages from SD rats showed restrained *L. amazonensis* growth, with intracellular amastigotes remaining low and only showing a slight increase at 72h postinfection (HPI). A considerable amount of NO was detected in the culture supernatant (Figures 1A,D). Nitric oxide (NO), produced by NOS2, has been shown to be the key antimicrobial agent found in macrophages, and its induction is critical in the control of many intracellular pathogens (Li et al., 2012). Therefore, we tested whether NO is important for conferring resistance to *Leishmania* infection in rat macrophages by inhibition of NOS2 activity with a specific inhibitor aminoguanidine (AG; Griffiths et al., 1993). When NOS2 was inhibited by AG, NO was not detected and parasite replication increased significantly, suggesting that NOS2 activity is indispensable in resistance (Figures 1A,D). To further dissect the role of NOS2, we generated an *Nos2* gene knockout SD (*Nos2*^−/−^) rat using transcription activator-like effector nuclease technology (TALENs; Tesson et al., 2011). TALENs resulted in a two-nucleotide deletion in the open reading frame of the *Nos2* gene (Supplementary Figure 1A). Disruption of the *Nos2* gene was demonstrated by the absence of NOS2 proteins in naïve or activated peritoneal macrophages and the absence of NO production in the culture supernatant of the activated macrophages (Supplementary Figures 1B,C), while arginase activity showed no significant difference between the wild-type and *Nos2*^−/−^ macrophages (Figure 1F). Macrophages from *Nos2*^−/−^ rats showed a significant increase in parasite burden compared to the macrophages from the wild-type rats at 72h postinfection (HPI; Figure 1C). More importantly, they failed to eliminate amastigotes even in the presence of IFN-γ, unlike the macrophages from the wild-type rats (Figures 1B,C). These observations clearly demonstrate that the parasite growth in NOS2 inhibited wild-type or *Nos2*^−/−^ macrophages was caused by the lack of NO production (Figure 1). The slight reduction in the parasite burden in the *Nos2*^−/−^ rat macrophages after IFN-γ activation indicates that NO-independent killing of *L. amazonensis* might exist. However, it must play a minor role, since most of the parasites remain intact, indicating that NO is the predominant leishmanicidal effector in rat peritoneal macrophages.

### Rats Lacking NOS2 Lose Innate Resistance to *L. amazonensis* Infection and Develop Progressive Lesions

**Figure 2:**
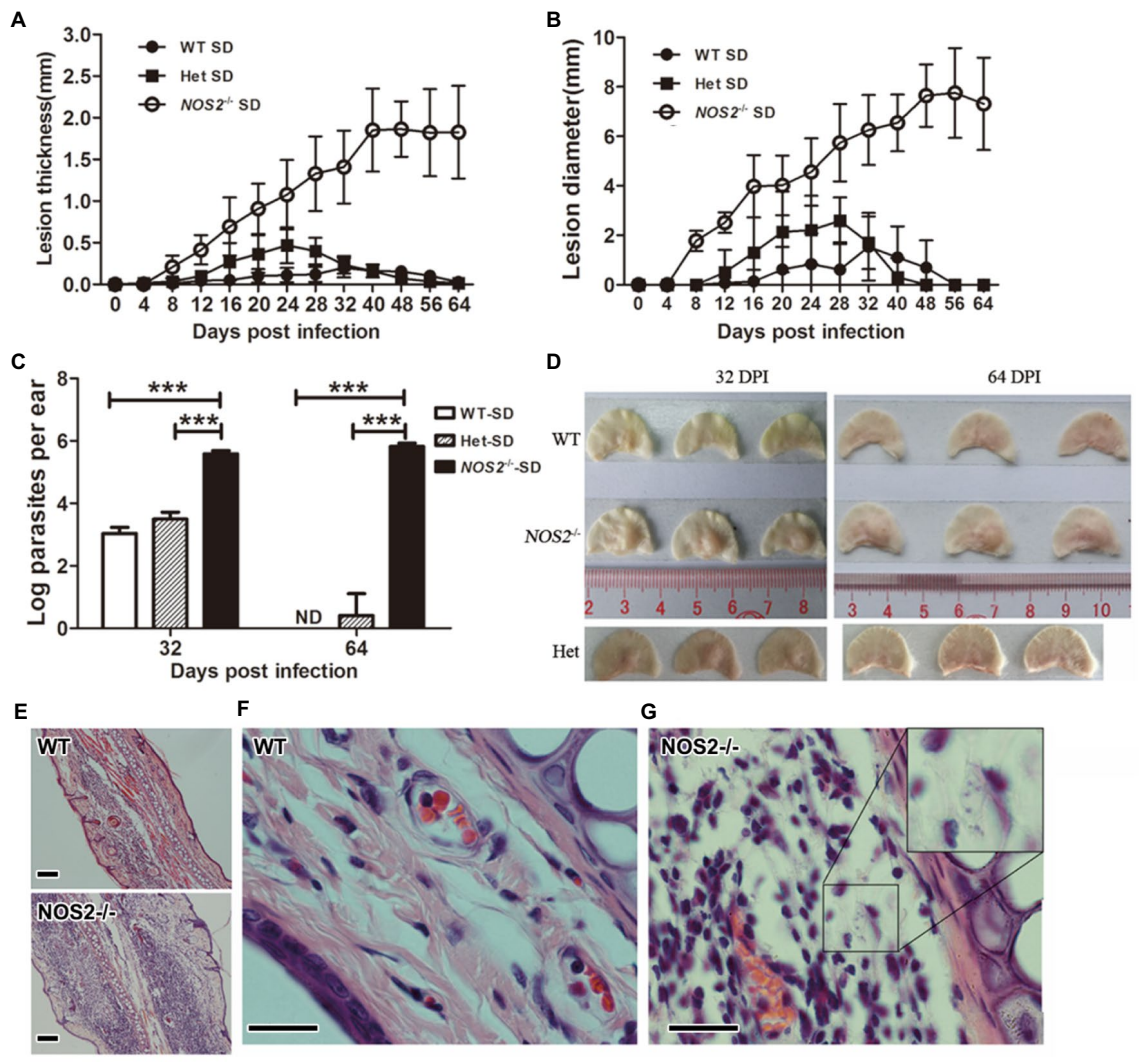
Rats deficient in NOS2 are susceptible and parasites were not controlled. Wild-type (WT), heterozygous (Het; *N*=12), and *Nos2*^−/−^ (*Nos2*^−/−^; *N*=12) SD rats were subcutaneously infected with 10^7^l*. amazonensis LV78* in the left ear. Lesion thickness **(A)**, and diameter **(B)** were shown as mean±SD. 0–32days postinfection (DPI), *N*=12; 40–64 DPI, *N*=6. The parasite load **(C)** in the lesion was determined by limiting dilution assay (*N*=3, mean±SD). The outlook of representative infected ears **(D)**. **(E–G)**, HE-stained sections of infected ear at 32 DPI. Inset in **G** shows multiple amastigotes. Scale bar, 20μm. ND: not detectable (<20). ^***^ indicates a significant difference (*P* ﹤ 0.001).

The above results demonstrate the critical role of NOS2 for rat macrophage resistance against *L. amazonensis* infection *in vitro*. We employed a rat ear cutaneous infection model to further investigate the role of NOS2 *in vivo*. Wild-type (WT), heterozygous (Het), and *Nos2*^−/−^ SD rats (*Nos2*^−/−^) were subcutaneously infected with 10^7^ stationary phase promastigotes of *L. amazonensis*. Interestingly, the results showed that all the *Nos2*^−/−^ SD rats were highly susceptible to *L. amazonensis* infection compared to their wild-type SD and Het counterparts. In all *Nos2*^−/−^ rats, lesions began to appear at 8days postinfection (DPI). Afterward, the lesions became progressively worse as shown by an increase in thickness and diameter. The increase in lesion pathology (thickness and diameter) occurred rapidly from 8 to 40 DPI, then levelled off, and did not show any sign of recovery (Figures 2A,B). In contrast to mouse strains and *Nos2*^−/−^ rats, the resistance to infection in wild-type SD rats was demonstrated by observing only a temporary mild swelling or even absence of swelling, in some animals, during the course of the experiment. At the end of the experiment (56–64 DPI), no lesions were found in any WT rats. Het rats developed moderate lesions at the early stages of infection but were also able to heal afterward. Hematoxylin-Eosin (HE)-stained sections of ear lesions at 32 DPI revealed that, in the *Nos2*^−/−^ rat, the cutaneous tissue in the thickened ear was dominated by an increased infiltration of inflammatory cells. However, unlike observations in mice (Vasconcellos and Sotto, 1997), granulomas were not observed at the site of the infection in rats even though there was extensive cell infiltration. Furthermore, the general structure of the ear was not affected. Some recruited cells in the *Nos2*^−/−^ rats harbored numerous amastigotes, indicating their inability to kill parasites (Figures 2D,E,G). In sharp contrast, the structure of the dermal tissue from the ear of wild-type rats remained either normal at 32 DPI or with a few cells infiltrating if mild swelling was present. However, we were unable to observe any amastigotes in the sections of WT ears, indicating that most of the injected parasites were eliminated (Figures 2D,F). The parasite burden of infected ears was estimated at 32 and 64 DPI, by a limiting dilution assay. The parasite load in lesions was much higher in *Nos2*^−/−^ rats than in the wild-type and Het rats. At 32 DPI, parasite load in the *Nos2*^−/−^ rats was almost 330-fold greater than that observed in WT rats. Furthermore, the parasite burden declined rapidly to an undetectable point in WT rats at 64 DPI while it persisted in *Nos2*^−/−^ rats. The parasite load in the Het rats was higher than that observed for WT rats at 32 DPI (*p*=0.027), but they were almost eliminated at 64 DPI (Figure 2C). The above results demonstrate that NOS2 was also indispensable for rat innate resistance to *L. amazonensis in vivo*.

### Rats Lacking NOS2 Present Normal *Leishmania*-Induced Specific Immune Responses After Infection

**Figure 3:**
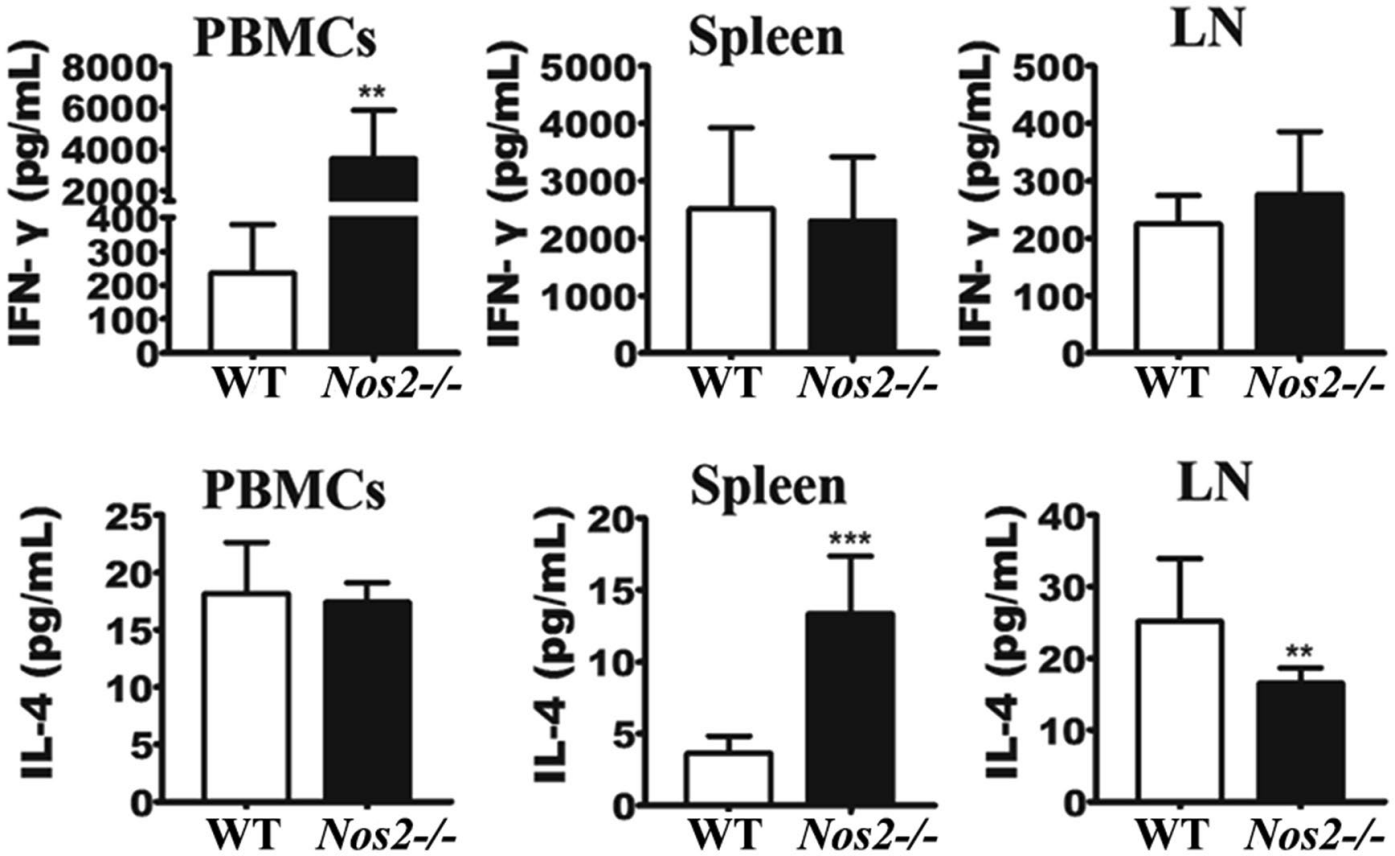
IFN-γ and IL-4 production in rat immune cells stimulated with SLA. 2×10^5^ PBMCs, LN, and spleen cells were isolated and treated with 50μg/ml soluble *Leishmania* antigens (SLA); IFN-γ and IL-4 production were determined from culture supernatants at 48h post-treatment (*N*=6, mean±SD). ^**^ indicates a significant difference (0.001﹤*P*﹤0.01);^***^ (*P*﹤0.001).

The above results have shown that NOS2 plays a pivotal role in rat resistance against *Leishmania* infection, both *in vitro* and *in vivo*. Since NO has been shown to participate in both antimicrobial activity and immune regulation in mice (Taylor-Robinson et al., 1994; Diefenbach et al., 1999), it is important to know whether rats deficient in NOS2 are capable of mounting an effective immune response toward *Leishmania*. Naïve *Nos2*^−/−^ rats did not show any observed abnormalities in the differentiation of CD4^+^ or CD8^+^ T-cell subsets (Supplementary Figure 2), as has previously been reported for NOS2-deficient mice (Wei et al., 1995). Both wild-type and *Nos2*^−/−^ groups of rats were each subcutaneously infected with 10^7^l*. amazonensis via* the dermal tissue in the ear. Then, peripheral blood mononuclear cells (PBMCs), splenocytes, and mesenteric lymph node (LN) cells were isolated 32 DPI and stimulated with soluble *Leishmania* antigens (SLA). Culture supernatants were assayed for IFN-γ and IL-4 production by ELISA. Splenocytes and LN cells from both WT and *Nos2*^−/−^-infected rats produced comparable amounts of IFN-γ, while a significantly higher concentration of IFN-γ in PBMCs was detected in the *Nos2*^−/−^ group than that in the WT group (*p*=0.0014; Figure 3), indicating that *Nos2*^−/−^ rats are able to mount a strong *Leishmania*-specific immune response. The higher level of production of IFN-γ in *Nos2*^−/−^ rats is presumably due to the persistence of greater numbers of parasites in the ear dermis, thus eliciting more parasite antigen to be recognized by the immune cells. IL-4 was also detected in both *Nos2*^−/−^ and WT rat cells.

**Figure 4:**
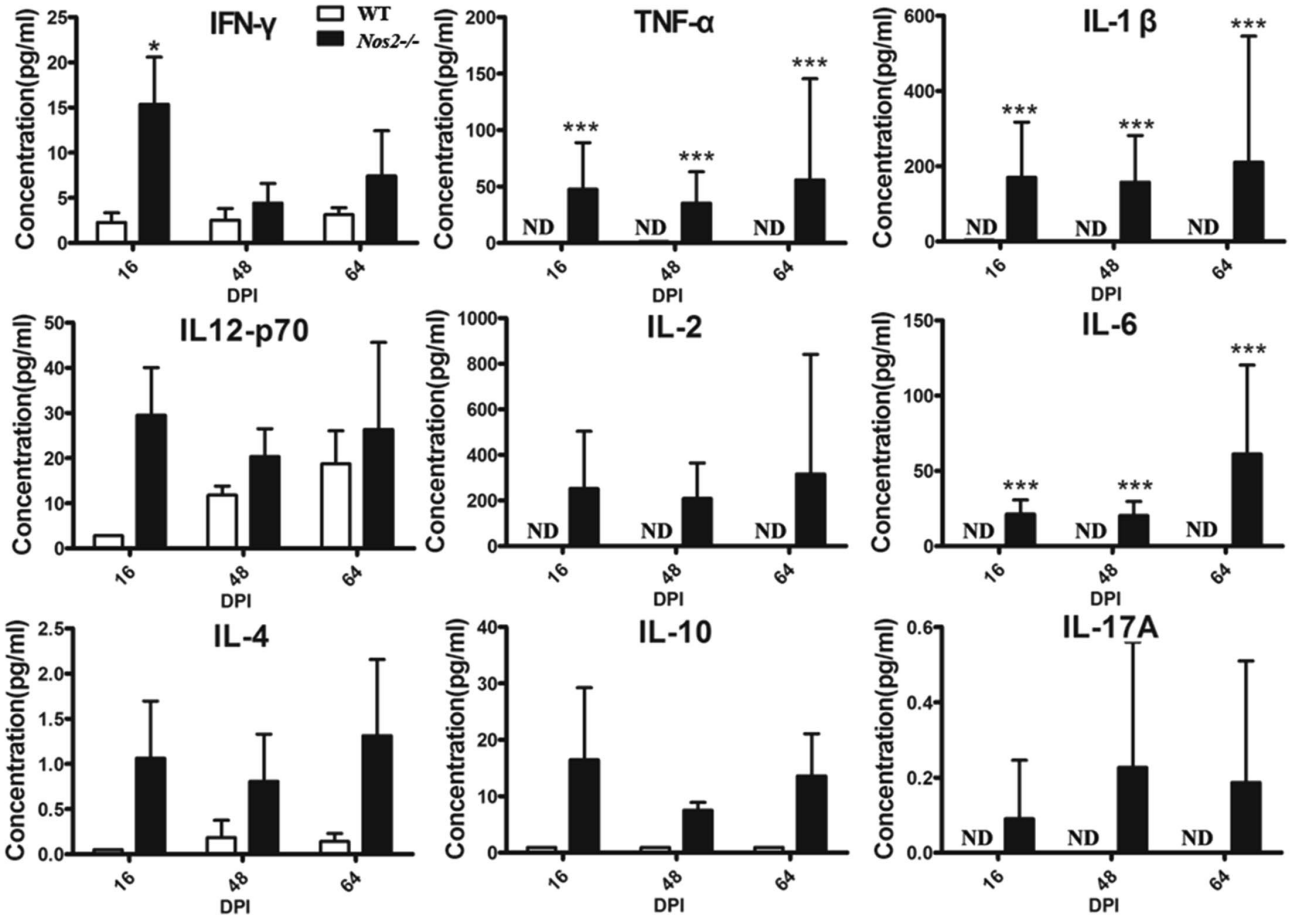
Serum cytokine profiles of rats infected with *Leishmania amazonensis*. The serum of *Nos2*^−/−^ and wild-type rats were collected at 16, 48, and 64days postinfection (DPI). Cytokine levels were determined by multiplex Luminex (*N*=3, mean±SD). ND: not detectable. ^*^indicates a significant difference (0.01﹤*P*﹤0.05);^***^ (*P*﹤0.001).

Serum cytokines profiles were measured to better understand the immune status in these rats. Inflammatory cytokines were significantly higher in *Nos2*^−/−^ rats but low to undetectable in WT rats (Figure 4). IFN-γ, TNF-α, IL-12, IL-1β, and IL-6 are important pro-inflammatory mediators during tissue inflammation. An increase in these cytokines was observed in the *Nos2*^−/−^ rats at all the time points tested, indicating that tissue inflammation persisted and was intense. Furthermore, this further supports the evidence that the *Nos2*^−/−^ rats were capable of mounting a specific immune response to *Leishmania*.

### Adoptive Transfer of Wild-Type Bone Marrow-Derived Macrophages to *Nos2*^−/−^ Rats Delayed Early Lesion Formation and Reduced Parasite Burden

**Figure 5:**
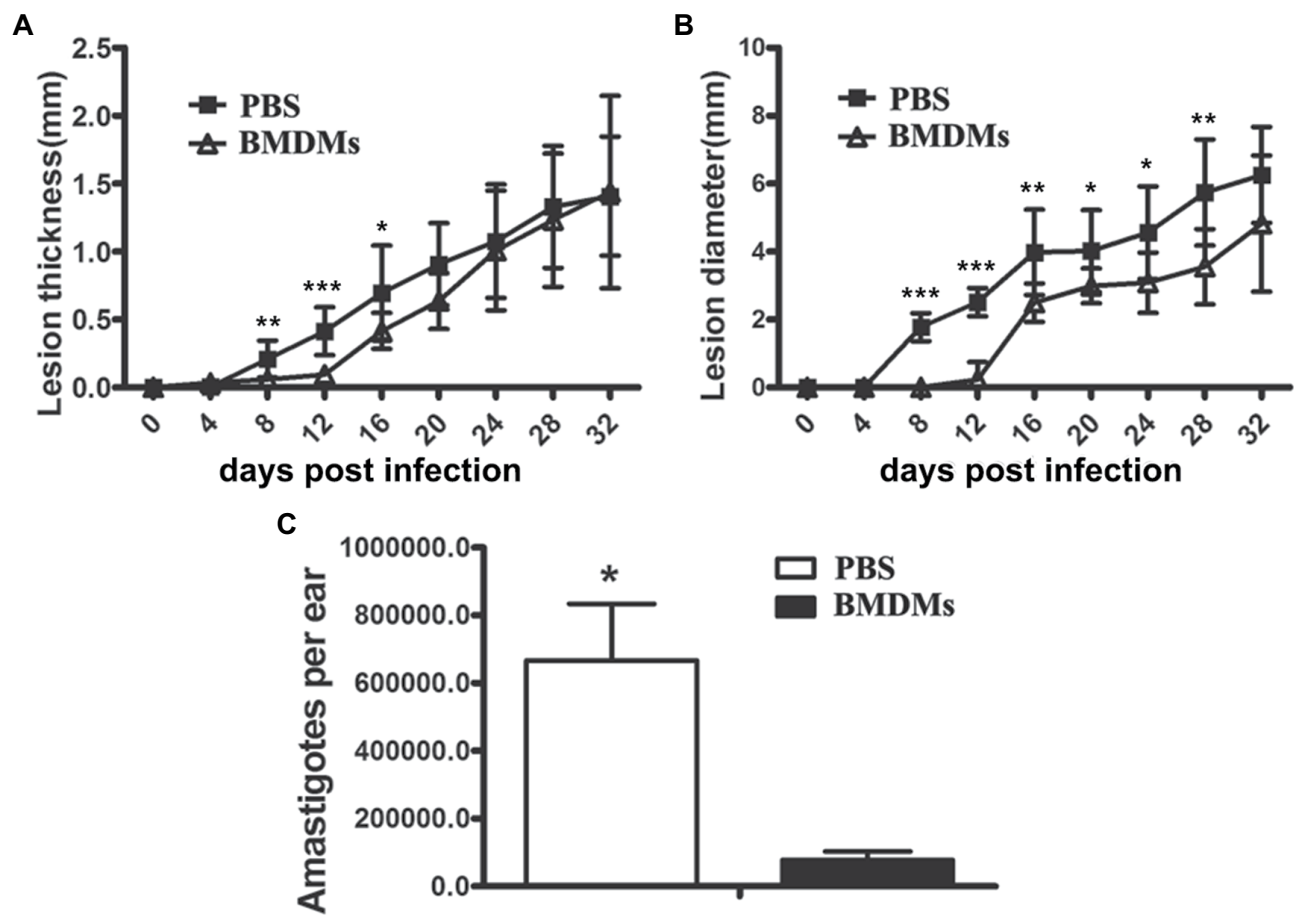
Adoptive transfer of wild-type macrophages to *Nos2*^−/−^ rats delayed lesion formation and reduced parasite load. Bone marrow cells were isolated from wild-type rats, cultured and differentiated to macrophages (BMDMs) in 30% L929 cell conditioned medium. *Nos2*^−/−^ rats (*N*=5) received 3, 5, 5, 8×10^6^ wild-type rat BMDMs at 0, 8, 16, and 24days postinfection, respectively. Lesion thickness **(A)** and diameter **(B)** were measured every 4days. **(C)** Parasite burden (mean±SD) on 32 DPI was determined by Limiting dilution assay. ^*^ indicates a significant difference (0.01 ﹤ *P* ﹤ 0.05); ^**^(0.001 ﹤ *P* ﹤ 0.01); ^***^(*P* ﹤ 0.001).

The above results provided strong evidence for the important role of NOS2 against *Leishmania* infection in rats. Since the immune regulatory network in *Nos2*^−/−^ rats is unimpaired, we therefore sought to investigate the role of macrophages in the rat model by adoptive transfer of BMDM from WT rats to the *Nos2*^−/−^ rats. In the *Nos2*^−/−^ rats that received WT BMDMs, the onset of the lesion development was delayed and shifted from 8 DPI to 16 DPI (Figure 5). Meanwhile, both the lesion thickness and diameter were significantly lower than in the untreated group prior to 20 DPI. However, lesions in the treated group developed progressively at a later time point (16–32 DPI). The lesion thickness increased to the same level as the untreated group but the diameter was still lower at 32 DPI. Nevertheless, the *Nos2*^−/−^ rats receiving wild-type BMDMs harbored significantly less parasites in lesions compared with the untreated group. These results provided further evidence that macrophages with NOS2 function play an important role in the control of parasites *in vivo*.

## Discussion

There is a basic question as to why rats are much more resistant to *Leishmania* spp. infection than mice? Host specificity is a commonly used answer. But what is the mechanism underpinning such specificity? So far as we know, before we started our study, this remained as a black box. To address the question of why rats are much more resistant to pathogen infection than mice, our group previously reported that the high basal NOS2 but low basal arginase activity in rat peritoneal macrophages were linked to their resistance to *Toxoplasma* infection (Li et al., 2012). Furthermore, results from a comparison of rat alveolar macrophages (sensitive to *Toxoplasma* infection) with rat peritoneal macrophages (resistant to *Toxoplasma* infection) showed that this difference was correlated with low and high NOS2 expression, respectively (Zhao et al., 2013). Additionally, differing levels of resistance and susceptibility to *T. gondii* in different inbred rat strains were correlated with the expression of NOS2 (Gao et al., 2015). More recently, we found that nitric oxide plays a key role in blocking the development of the human parasite Schistosoma japonicum in the rat (Shen et al., 2017). However, arguments still exist about the distinctive differences between *Toxoplasma* and *Leishmania* infection. *Toxoplasma* can infect almost all cells with a nucleus, while *Leishmania* almost only infects phagocytes but predominantly macrophages.

To exclude the possibility that other mechanisms, rather than L-arginine metabolism, play the key functional role in host specificity, our study used an even more simple model to address this question. As *Leishmania* infects only macrophages, a *Nos2* gene knockout model system can be used to investigate the role of NOS2 in the resistance to *Leishmania* infection in rats. The importance of macrophage NOS2 in rat resistance to *Leishmania* has been demonstrated both *in vitro* and *in vivo* by the following evidence: 1. Enhanced intracellular survival and replication of amastigotes in NOS2 inhibited wild-type macrophages or *Nos2*^−/−^ macrophages, 2. inability to eliminate amastigotes after activation with IFN-γ in *Nos2*-deficient macrophages, 3. subclinical infection and parasite elimination in wild-type rats compared with progressive lesion development and persistent parasitemia in *Nos2*^−/−^ rats, even though the immune response was unimpaired, and 4. transfer of macrophages collected from wild-type rats into *Nos2*^−/−^ rats delayed lesion formation and reduced parasite load.

As our data mentioned above clearly show, after infection with an invading pathogen, L-arginine metabolism is key to shaping macrophage function and determines the progression of the disease. Macrophages are major players in the first line of innate defense against many pathogen infections. It is, therefore, fundamental to understand the differences in arginase metabolism in macrophages from various hosts and even between different human populations. Data from Mills and colleagues revealed that macrophages from different mouse strains varied in arginine metabolism in response to immunological stimuli, such as lipopolysaccharide (LPS) or IFN-γ; thus, they classified macrophages that predominately express NOS2 or arginase pathways as M1 or M2 macrophages, respectively (Mills et al., 2000). However, more striking differences exist between mouse and rat macrophages. When rats are used as a reference, all mouse macrophages, in different inbred lines, were highly polarized toward the M2 state and were characterized by prominent arginase activity and undetectable NOS2 activity (Li et al., 2012). In contrast, naïve macrophages from rat strains were uniformly polarized toward the NOS2 pathway, where arginase activity was low, and thus, they are polarized toward M1 state. More importantly, this polarization is independent of cytokine or microbial product stimulation (Li et al., 2012). Interestingly, other than in the naïve state, rat macrophages are also more sensitive to exogenous stimuli than mice macrophages, as they are readily activated at a lower LPS concentrations or produce higher amounts of NO when treated with the same concentration of LPS (Kmonickova et al., 2007). Differential tendencies in arginine utilization were also observed in macrophages during infection. The inducibility of NOS2 activity by LPS was found to be much higher in rat macrophages than mouse macrophages infected with *L. amazonensis*. Infected rat macrophages produced substantial amounts of NO at low LPS concentrations, while mouse macrophages required a much higher LPS concentration but only generated limited amounts of NO (Supplementary Figure S3). These findings have pointed to distinctive differences between macrophage potency in generating NO. This may explain why rats, but not mice, are able to control subclinical infections of *L. amazonensis* and achieve sterile cure. Therefore, this potentially provides a strong mechanistic link to the explanation behind the concept of host specificity. Besides the pivotal role of NOS2, the role of arginase in rat resistance against *Leishmania* infection remains to be defined. Interestingly, arginase activity is naturally low in rats but high in mice macrophages (Li et al., 2012). High arginase and subsequent polyamine biosynthesis favor parasite survival and replication, and this is a biomarker for active disease status in VL (Iniesta et al., 2002; Kropf et al., 2005; Abebe et al., 2013). Furthermore, evidence has demonstrated that inhibition of arginase activity reduces *Leishmania* growth and consequent disease development in infected murine hosts (Iniesta et al., 2001, 2005). Taken together, rat macrophages have a propensity to activate the NOS2 pathway during *L. amazonensis* infection with high NO output accompanied by limited arginase activity. This then creates a highly detrimental microenvironment for *Leishmania* survival resulting in innate resistance. The disruption of the *Nos2* gene in the knockout rat, and subsequent susceptibility, strongly supports this conclusion. Therefore, we conclude that the host specificity to parasites, at least for *Leishmania* in rat and mouse models, is mainly due to differences in L-arginine metabolism and polarization toward NOS2 activity.

In our rat model, the deletion of two nucleotides in the *Nos2* gene of rats reversed its resistance to *Leishmania* infection. In view of the long-term co-evolution of host and parasite, the mutation of a single gene is likely to be common and could give rise to different populations that show diversity in gene function. The maintenance of a crucial gene that is responsible for parasite resistance, such as NOS2, is an important selective pressure for the evolution of the host. Any changes in the regulatory pathways that enhance the function of these genes would promote host survival and expansion, especially when the infectious agent is both fatal and present at high prevalence. Rats are, therefore, an extraordinary host that is highly resistant to many pathogen infections. In contrast, gene mutations that weaken or abrogate these genes would favor parasite adaptation to the host, such as occurs in mice and hamsters that are susceptible to infection. The evolution of the *Nos2* gene and its regulatory network in different hosts is extremely interesting, and the importance of this gene is, at least, demonstrated in the rat and mouse. Genomic analysis in the rat has shown that rat-specific genes related to immunity have appeared through an expansion of gene families, which is not seen in the mouse (Gibbs et al., 2004). Further analysis through comparative genomic analysis would be valuable in identifying other genes that confer innate resistance.

In humans, the involvement of NOS2 in protection against leishmaniasis has been demonstrated by the finding that higher NOS2 expression correlates with fewer parasites in localized CL, while low NOS2 expression correlates with high parasite burden in diffuse CL (Qadoumi et al., 2002). In another example, the Tat protein, produced by HIV-1, appears to interfere with protein kinase R (PKR) activity which downregulates NOS2 expression in macrophages derived from healthy human donors and leads to consequent increase in intracellular growth by *L. amazonensis* (Vivarini Ade et al., 2015). Additionally *L. amazonensis*, itself, may be responsible for down regulation of NOS2 in human and mouse macrophages *via* the NF-κβ pathway (Calegari-Silva et al., 2015). Furthermore, NOS2 has been shown to participate in a variety of other human diseases (Kroncke et al., 1998). The question as to whether NOS2 plays a dominant role in human disease is under debate (Albina, 1995; Jungi et al., 1996). There has been some evidence that higher NOS2 activity in humans correlates with protective immunity to infections (Nicholson et al., 1996). For example, in endemic areas with malaria, higher resistance to the disease is present in people with higher NO production in macrophages (Anstey et al., 1996). Thus, by comparison with species differences in rodents, genetic control of differences in NOS2 activity in human populations might also exist. A higher NOS2 activity during infection might also confer protection. Asymptomatic infections of *Leishmania* in humans have been reported. In infections caused by *L. infantum*, for example, the majority of individuals infected are asymptomatic (Michel et al., 2011). Little is known about the differences in immune status between symptomatic and asymptomatic patients with leishmaniasis. Investigations into the possible involvement of NOS2 might enhance insight into the evolution of the *Nos2* gene function and population-related immunity. Based on our findings in the rat model, we suggest that the status of L-arginine metabolism in macrophages reflects host immune function against pathogen infection. High NOS2 activity is a marker for protective response in resistant hosts, while high arginase activity is associated with a suppressed immune response in susceptible hosts.

In summary, the results described here show that a distinctive L-arginine metabolism featuring high NOS2 activity in rat macrophages is responsible for resistance to *Leishmania* infection. Our data indicate that in addition to the extrinsic factors that shape macrophage function, rat macrophages independently express an innate resistance phenotype to infection. Such inherent properties of the innate immune system are linked to host specificity for parasite infections in various hosts as demonstrated here between rats and mice. This linkage could have important implications for the development of immunotherapy by targeting L-arginine metabolism in leishmaniasis and, perhaps, many other pathogen infections.

## Data Availability Statement

The original contributions presented in the study are included in the article/**Supplementary Material**, and further inquiries can be directed to the corresponding authors.

## Ethics Statement

The animal study was reviewed and approved by Institutional Review Board for Animal Care at Sun Yat-Sen University.

## Author Contributions

Z-RL, Y-FC, C-YW, S-FY, and D-HL designed the experiment. Y-FC, S-FY, NW, JiS, JuS, J-MG, and Y-ZW did the experiments. Z-RL, Y-FC, S-FY, C-YW, D-HL, and GH analyzed the data. Y-FC, Z-RL, D-HL, and GH wrote the manuscript. All authors approved the final version of the manuscript.

## Funding

This work was supported by grants (31720103918 and 31672276) from the National Natural Science Foundation of China to Z-RL, and the Natural Science Foundation of Guangdong Province (2018A030313187) to Y-ZW.

## Conflict of Interest

The authors declare that the research was conducted in the absence of any commercial or financial relationships that could be construed as a potential conflict of interest.

## Publisher’s Note

All claims expressed in this article are solely those of the authors and do not necessarily represent those of their affiliated organizations, or those of the publisher, the editors and the reviewers. Any product that may be evaluated in this article, or claim that may be made by its manufacturer, is not guaranteed or endorsed by the publisher.
