# Supplementary material for: Innate Resistance to Leishmania amazonensis Infection in Rat Is Dependent on NOS2
Source: Front Microbiol. 2021 Oct 29;12:733286. doi: 10.3389/fmicb.2021.733286 (PMC8586549; doi:10.3389/fmicb.2021.733286)
Supplement: Supplementary file 1 [file Data_Sheet_1.DOCX]

Supplementary Material

# Supplementary Figures and Tables

## Supplementary Figures


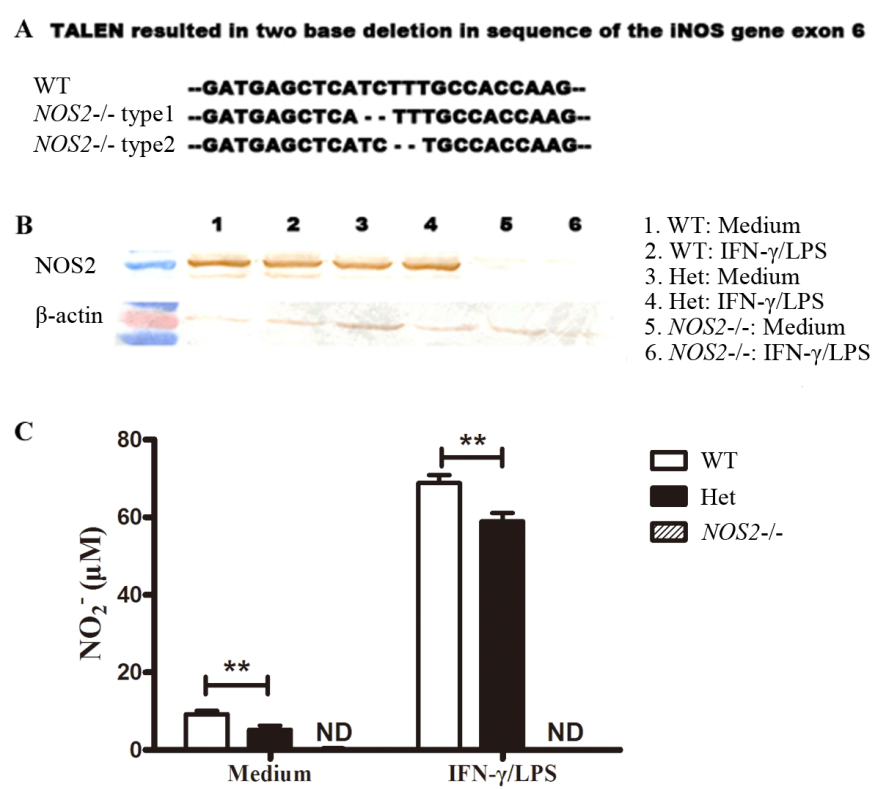


**Supplementary Figure 1.** **Generation and identification of the NOS2 knockout rat.** (**A**) Knockout mutants of the inducible nitric oxide synthase (NOS2) in SD rats were produced by an efficient site-specific gene modification technique called TALENs. A pair of TALENS targeting Exon 6 of SD rat *Nos2* gene were designed. (**B**) Peritoneal macrophages from different wild type (WT) rats, heterozygous (Het) rats and knockout (*Nos2*^-/-^) rats were stimulated for 24 hours with or without LPS (100 ng/ml) plus IFN-γ (50 ng/ml). Macrophages were lysed and NOS2 and β-actin, in lysis supernatants, were analyzed by western blotting. (**C**) Macrophages were stimulated for 24 hours with or without IFN-γ (50 ng/ml) plus LPS (100 ng/ml), the production of nitric oxide in culture supernatants was determined by the Griess reaction.


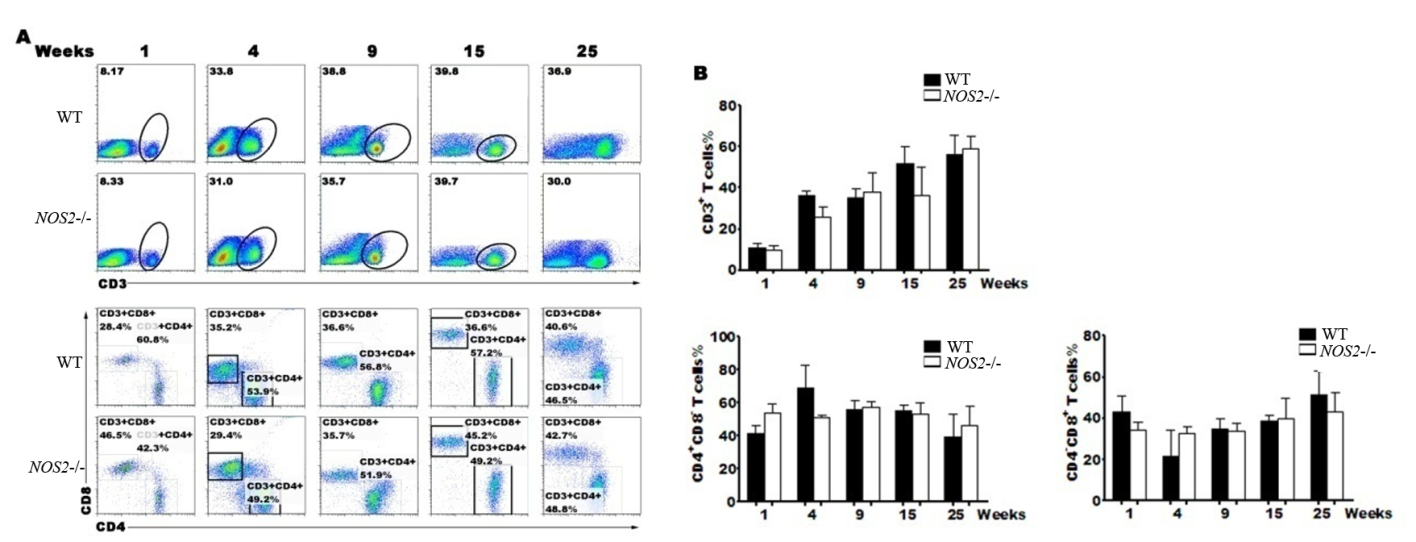


**Supplementary Figure 2.** **Phenotypic characterization of T lymphocytes isolated from the spleens of WT and KO rats.** (**A**) Mononuclear cells were isolated from spleens of 1, 4, 9, 15 and 25 week old WT and *Nos2*^-/-^ rats; immunofluorescent anti-bodies for CD3, CD4 and CD8 were used to analyze the T lymphocyte properties. Representative dot graphs are shown. (B) Proportions of CD3^+^, CD3^+^CD4^+^ and CD3^+^CD8^+^ T cells in the total lymphocyte population are shown.


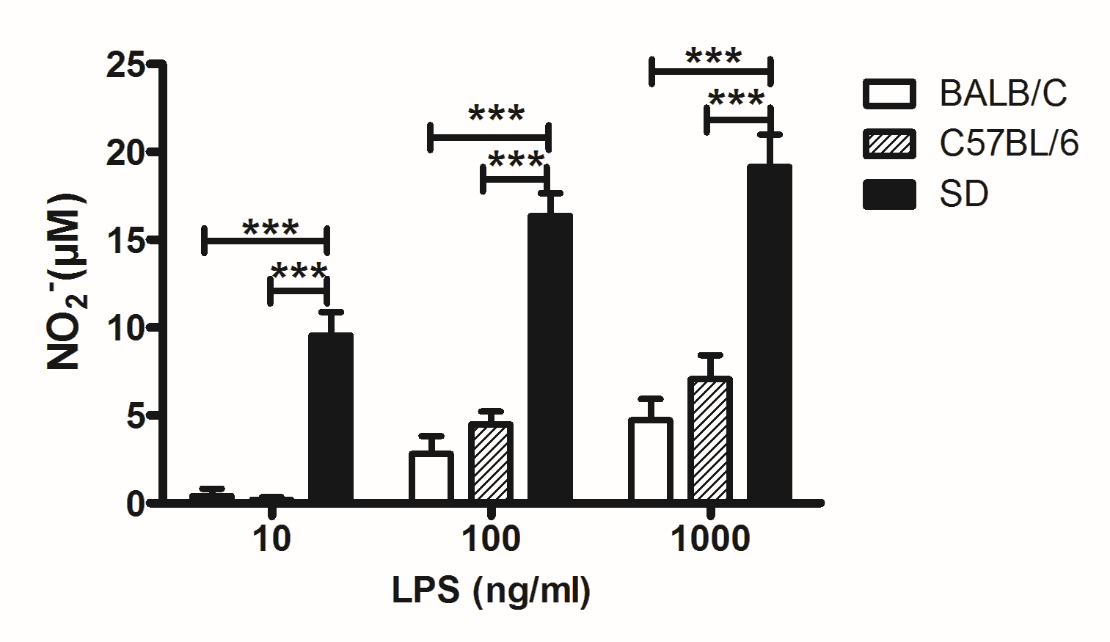


**Supplementary Figure 3.** **Production of NO in mouse and rat macrophages infected with *L. amazonensis* after LPS treatment.** Peritoneal macrophages from BALB/C, C57BL/6 mice and SD rats were infected with *L. amazonensis* promastigotes at a parasite to macrophage ratio of 5:1 for 6 hours, after which the extracellular parasites were washed away and replenished with fresh medium containing 10, 100 or 1000 ng/ml LPS. NO was determined from culture supernatant 24 hours post treatment. *** indicates a significant difference (*P*﹤0.001).
